# Development of a Survey of Sunscreen Use and Attitudes among Adults in Two Coastal States, 2019

**DOI:** 10.3390/ijerph19052677

**Published:** 2022-02-25

**Authors:** Karen Glanz, Pui L. Kwong, Jade Avelis, Kevin Cassel

**Affiliations:** 1 Perelman School of Medicine, University of Pennsylvania, Philadelphia, PA 19104, USA; luikwong@pennmedicine.upenn.edu (P.L.K.); jade.avelis@pennmedicine.upenn.edu (J.A.); 2 School of Nursing, University of Pennsylvania, Philadelphia, PA 19104, USA; 3 Population Sciences in the Pacific, University of Hawaii Cancer Center, Honolulu, HI 96813, USA; kevin@cc.hawaii.edu

**Keywords:** skin cancer, sunscreen, prevention education, policy, public health

## Abstract

Skin cancer is the most common form of cancer in the United States, and regular use of broad-spectrum sunscreens can prevent skin cancer. However, a new law in Hawaii that limits sunscreen choices due to the belief that some UV (ultraviolet) filters may damage coral reefs may reduce sunscreen use and increase skin-cancer risk. Because of this, there is a need for measurement tools to help understand consumer behavior and determinants of sunscreen purchase and use. The objectives of this study were (1) to test new questionnaire measures relevant to the Hawaii Sunscreen Ban; and (2) to assess adults’ knowledge, attitudes, and habits related to sunscreen in two other coastal states. This survey of adult residents of California and Florida was conducted in the summer of 2019. Newly developed scales addressed beliefs about effects of sunscreens on aquatic/marine environments and awareness of the Hawaii sunscreen ban. Respondents completed the survey twice to evaluate the test–retest reliability. Respondents (*n* = 162) were mainly female, White, and college-educated. New scales had moderate-to-high internal consistency and high test–retest reliability. Sunscreen use was high, sunburn was common, and knowledge and attitudes about sunscreen were modest. Most respondents did not know the specifics of the Hawaii Sunscreen Ban. In multivariate models, significant predictors of sunscreen use were being older, female, and having higher sunscreen knowledge. Sunscreen beliefs were not significantly associated with sunscreen use or sunburn. The findings support the use of the newly developed survey and suggest that more education about sunscreen and sunscreen ingredients is needed.

## 1. Introduction

Skin cancer is the most common form of cancer in the United States, and the incidence of melanoma continues to rise [1]. Recent trials support the efficacy of broad-spectrum sunscreen to prevent skin cancer and photoaging [2]. Sunscreen is one of the most-often used skin-cancer-prevention strategies, but it is usually or always used by about one-third of adults [3] when outdoors [4]. An estimated 11–38% reduction in melanomas by 2031 would occur in the US White population with increased regular sunscreen use [5]. However, a new law that limits sunscreen availability and choice may lead to lower use of sunscreen and increased risk of skin cancer.

In May 2018, the Hawaii State Legislature passed a law banning the sale and distribution of sunscreens containing oxybenzone or octinoxate in the state [6] (Hawaii Sunscreen Ban), due to concerns about these chemicals’ potential to damage coral reefs [7,8]. The law was passed despite the remaining controversies and questions about the quality and conclusions of emerging research about the effects of chemical sunscreens, i.e., those with “organic UV filters”, on coral reefs and the marine environment [9]. Notably, oxybenzone and octinoxate are the two most common active ingredients, or UV filters, in over-the-counter sunscreen products. The law prohibits sales of “chemical sunscreen” products that include these two UV filters in the state, but these products can be brought in when traveling or purchased online [6]. While there are numerous available UV filters [10], oxybenzone is one of the few filters that blocks most UVA (ultraviolet A) and UVB (ultraviolet B) radiation, and it is used in products that consumers tend to rate most highly [11,12].

With this law going into effect in Hawaii January 2021, and similar laws under consideration or implemented elsewhere, including the US Virgin Islands [13], Palau, Bonaire, Aruba, Mexico, and Thailand [14,15], it is important to understand the public’s knowledge, attitudes, and habits related to sunscreen and to develop new and relevant questionnaire measures relevant to the “sunscreen bans”. Available and widely used measures of skin-cancer-prevention behaviors and their determinants have examined skin-cancer knowledge, norms, and attitudes [16,17], but there is a need for additional new measures of determinants of sunscreen choice and beliefs about whether there are possible harms of sunscreen to marine environments and humans. This study was conducted both to (1) develop and pilot test those new measures and (2) to assess adults’ perceptions and behaviors and their association with sunscreen use. The survey was conducted in Florida and California, given their substantial coastlines and high levels of sun exposure, as well as the availability of a convenience sample of adults enrolled in the ResearchMatch registry.

## 2. Materials and Methods

Survey Methods. The survey was conducted in July–August 2019. It was administered on the Qualtrics platform, and recruitment took place through ResearchMatch.org, an online registry of research volunteers (www.researchmatch.org (accessed on 12 November 2021)). Eligibility criteria included age 18 or older, could read English, capable of giving informed consent, and resided in California or Florida. We found that the ResearchMatch registry had only 12 adults in the state of Hawaii, so the inclusion of participants from other coastal states was a useful alternative for pilot testing this survey instrument.

ResearchMatch contacted eligible participants via email to invite them to take part in the study. Next, ResearchMatch released contact information for volunteers who indicated their interest to the research team, who emailed the volunteers a link to the online informed consent and survey (*n* = 265). All respondents (*n* = 162) were invited to complete the survey a second time, two weeks after the first survey, to evaluate the test–retest reliability.

**Figure 1:**
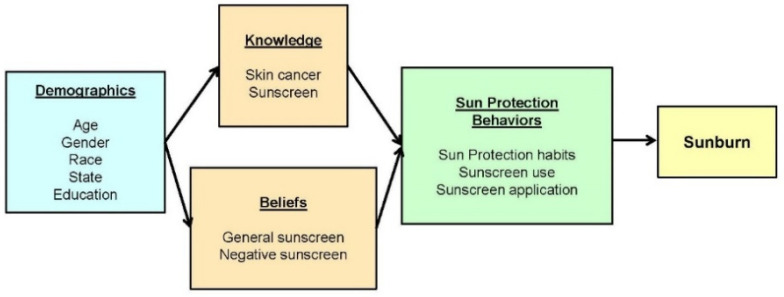
Conceptual model of correlates of sunscreen use and sunburn.

Measures. The survey took 10–15 min to complete. The survey examined correlates of sunscreen use and sunburn based on a conceptual model (Figure 1) that posits that demographic characteristics, knowledge, and beliefs contribute to sunscreen use and sunburn.

This survey tool used items from previous skin-cancer-prevention surveys developed by the authors and colleagues across the country [16] and adaptations of previously used measures of sunscreen purchase and use [17]. The items that had been previously developed and studied did not require validation. New measures were created for this survey to assess awareness and knowledge of the Hawaii sunscreen ban, beliefs about effects of sunscreens on aquatic/marine environments, and questions about where respondents buy sunscreen. These item were created based on key informational concepts related to sunscreen’s efficacy and the potential harms to people and the environment that have been raised in the media, and considered for legislation [18]. Specifically, the new measures included a general skin cancer knowledge score (6 items), sunscreen knowledge score (7 items), general sunscreen beliefs (8 items), a negative sunscreen beliefs score (5 items, about sunscreen’s harms to humans and marine environments), and awareness and knowledge of the Hawaii sunscreen ban (4 items). Knowledge items were scored as correct or incorrect, and the attitude questions were asked on Likert-type scales. Questions about sources for purchase of sunscreen were asked as categorical items. (The survey is available as a Supplementary File S1 to this article).

Statistical Analysis. Descriptive statistics were computed for all variables. Next, psychometric analyses were conducted for new composite measures, using Cronbach’s alpha to assess internal consistency [19,20] and test–retest reliability was assessed by using Kappa coefficients [21] by comparing responses across both survey occasions for personal characteristics and for six knowledge, beliefs, and behavior composite scales. The categories for acceptable ranges of the alpha and kappa coefficients were based on Nunnally [19] and Landis and Koch [21], respectively.

We conducted multivariate analyses to examine correlates of sunscreen use and sunburn based on the conceptual model (Figure 1) to assess the relative contribution of demographics, knowledge, and beliefs to sunscreen use and sunburn. The primary outcome variables were reported sunscreen use “usually/sometimes” when outdoors in warm weather and sunburn within the past year.

For these multivariate analyses, the GENMOD procedure was used to fit generalized linear models in logistic regression with binomial responses. There were 151 subjects included in these analyses. The multiple imputation procedure (imputation # = 20) with fully conditional specification method (FCS) was performed for missing data on some variables: notably, 39 were missing gender, and 11 were missing on sunscreen application scale. The MIANALYZE procedure was used to combine results of the analyses of imputations to generate valid statistical inferences. Models were fit by using SAS version 9.4 [22]. Results of multivariate models are shown as odds ratios (ORs) with the corresponding *p*-values. Odds ratio is used as a statistical outcome in logistic regression when the outcome is binomial to indicate the association between independent variables/covariates and an outcome. The result is the impact of each variable on the odds ratio of the observed event of interest when controlling for other variables in the model.

## 3. Results

### 3.1. Sample

Of the 265 volunteers sent a link to the survey, 162 (61.1%) completed the first survey; 53 people completed the second survey (34.6%). The sample was 75% female, 85% White, with a mean age of 52.8 (±17.5), and 72.2% college graduates. A total of 60.9% of respondents were from California, 32.3% from Florida, and 6.8% resided in another state (although they had registered for ResearchMatch while living in either Florida or California).

#### 3.1.1. Internal Consistency of New Scales and Test–Retest Reliability

Composite measures for new knowledge and belief scales all had moderate-to-acceptable internal consistency: sunscreen knowledge (seven items, alpha = 0.59), general sunscreen beliefs (eight items, alpha = 0.72), and negative sunscreen beliefs (five items, alpha = 0.76). Comparisons for test–retest reliability between surveys revealed no significant differences in personal characteristics and high correlations for all six composite measures (r = 0.73–0.89 or substantial to excellent for five scales, 0.57 or moderate for skin cancer knowledge; all *p* < 0.001).

#### 3.1.2. Behaviors, Beliefs and Knowledge

Table 1 shows results for sunburn and sun-protection behavior. A total of 53.1% reported usually or always using sunscreen when outdoors on a warm day, and sunscreen users applied sunscreen on more than half of their exposed body parts. A total of 58% reported one or more sunburn in the past year. Sunscreen-knowledge scores averaged 3.41 (s.d. 1.06) on a scale of 0 to 7, and both general sunscreen beliefs and negative sunscreen beliefs were near the middle of each scale. The most important features for sunscreen choice were broad spectrum (66.3%), SPF 15+ (63.0%) or 50+ (51.9%), and the ingredients (43.3%). While 20–25% indicated typically using a chemical or mineral sunscreen, the most common answer to these questions was “not sure”.

Only about one-third of respondents said they had heard about the sunscreen ban law passed in Hawaii, and knowledge of the law’s provisions was low (Table 2), with the most common response to three questions about the law being “don’t know” (56.2–71.6%) and most people (54.3%) answering none of the three questions correctly.

In multivariate models, significant predictors of sunscreen use were older age (OR = 1.03 (1.01–1.06)), being female (males OR = 0.34 (0.13–0.93)), and greater sunscreen knowledge (OR = 1.51 (1.06–2.16)) (Table 3). Correlates of avoiding sunburn (e.g., less sunburn) were older age (OR = 0.94 (0.91–0.96)), non-White race (OR = 0.22 (0.06–0.77)), and better sun-protection habits (OR = 0.33 (0.13–0.83)). Negative sunscreen belief scores, general sunscreen belief scores, and sunscreen knowledge were not significantly associated with sunscreen use or sunburn (Table 4).

## 4. Discussion

In this survey of mostly well-educated participants, a survey including newly developed scales to assess sunscreen attitudes and awareness of the Hawaii Sunscreen Ban showed good test–retest reliability and internal consistency for new scales. Although the internal consistency of the skin-cancer knowledge scale was lower than desirable, at 0.57, this scale has been found acceptable for use in previous studies [23] or has had low internal consistency in other studies [24]. We were unable to find an alternative measure of this construct. White middle-aged residents of California and Florida reported substantially higher sunscreen use than in the US as a whole, as is consistent with national rates of sunscreen use in different age groups [3]. However, sunburn was common, and knowledge and attitudes about sunscreen were in the middle ranges. However, while about one-third of the respondents had heard about the Hawaii Sunscreen Ban, very few knew the main provisions of the law.

Despite participants reporting that sunscreen ingredients are an important purchasing consideration, few could describe the specific type of ingredients in their sunscreens (mineral versus chemical). The main reported driver for sunscreen purchases was the products’ ability to provide effective protection from UV damage (broad-spectrum, SPF >15). Future studies are needed to help understand the impact of a ban on popular types of sunscreen on use of sunscreen, including the question of whether a possible decline in sunscreen use may be offset by greater use of protective clothing and shade.

Beliefs about sunscreen, including beliefs about their safety and potential harm on marine environments, were not predictors of either sunscreen use or sunburn in this study. This may be due in part to the respondents’ limited understanding about which types of UV filters in sunscreen have been found, in some studies, to pose a threat to marine environments. Put simply, if people do not hold strong beliefs, those beliefs are less likely to affect their behavior.

Limitations of our study include a well-educated and mostly White sample, which is racially/ethnically dissimilar from Hawaii residents. Moreover, our survey did not assess skin-cancer risk or history. Despite these limitations, this pilot study provides valuable preliminary data to support further inquiry in larger samples, and in residents and tourists in Hawaii and the US Virgin Islands. The new measures showed acceptable to very good psychometric characteristics, with the exception of the sunscreen-knowledge scale. The instrument will be very useful to assess the pre-ban to post-ban effects on sunscreen use in evaluations of the Hawaii Sunscreen Ban and its impact on Hawaii residents and visitors.

The survey measures reported here will be useful in obtaining more scientifically grounded information about sun safety and ultimately to develop appropriate and evidenced-based prevention education.

## 5. Conclusions

Our survey with newly developed scales to assess sunscreen attitudes and awareness of the Hawaii Sunscreen Ban showed good test–retest reliability and moderate-to-good internal consistency for new scales. White middle-aged respondents to the study had moderately favorable knowledge and attitudes about sunscreen and had heard about the Hawaii Sunscreen Ban, but most did not know the key features of the law.

Furthermore, few respondents knew whether the ingredients in their preferred sunscreens were chemical or mineral, which is the focus of concerns about sunscreens possibly being harmful to aquatic environments.

The rationale for the Hawaii Sunscreen Ban law is still being debated by scientists and clinicians [9,25,26]. This study provides a well-designed survey measure that can be used to learn more and evaluate changes in sunscreen attitudes and practices.

## Figures and Tables

**Table 1:** Respondent sun-protection practices, knowledge, and beliefs.

Characteristic	Total
	(*n* = 162)
Sun-Protection Behavior and Sunburn (Mean + Standard Deviation)	
% One or more sunburn in the last year	58.0% (94)
Sun-Protection Habits Index (M ± SD; range 1–5)	3.4 (0.6)
Use Sunscreen (M ± SD; range 1–5)	3.5 (1.2)
Seek Shade (M ± SD; range 1–5)	3.2 (0.9)
Wear Sunglasses (M ± SD; range 1–5)	4.0 (1.2)
Wear a Shirt With Sleeves (M ± SD; range 1–5)	3.1 (1.1)
Wear a Hat (M ± SD; range 1–5)	3.0 (1.3)
Limit Midday Hours in the Sun (M ± SD; range 1–5)	3.7 (1.0)
Sunscreen application score (M ± SD; range 1–9)	5.4 (2.3)
Knowledge and Beliefs	
Sunscreen Knowledge Score (M ± SD; range 0–7)	5.7(1.3)
General Sunscreen Beliefs Score (M ± SD; range 1–5) (positive direction)	2.8 (0.8)
Negative Sunscreen Beliefs Score (M ± SD; range 1–5) (negative direction)	2.8 (0.8)
Sunscreen Choice and Purchasing (*n* = 156; 6 do not purchase)	
Most important features when purchasing sunscreen (% very important/critical)	
Broad Spectrum	66.3%
SPF 15+	63.0%
SPF 50+	51.9%
Ingredients	43.3%
Usual sunscreen type	
Chemical (e.g., oxybenzone and octinoxate)	20.3% (60.1% not sure)
Mineral (e.g., zinc oxide and titanium dioxide)	24.8% (47.8% not sure)

**Table 2:** Awareness of the Hawaii Sunscreen Ban regulations.

Variable	Total
	(*n* = 162)
Heard about the 2018 law passed in Hawaii:	
Yes	34.0%
No	58.6%
Not sure	7.4%
Believe it is against the new law for stores to sell sunscreen containing oxybenzone or octinoxate in Hawaii (true)	
Correct	42.0%
Incorrect	1.9%
Don’t Know	56.2%
Believe it is against the law for consumers in Hawaii to purchase sunscreen containing oxybenzone or octinoxate on the internet (false)	
Correct	9.3%
Incorrect	19.1%
Don’t Know	71.6%
Believe visitors to Hawaii can bring sunscreen containing oxybenzone or octinoxate with them (true)	
Correct	17.3%
Incorrect	12.4%
Don’t Know	70.4%
Total number correct (of the three items above)	
Zero	54.3%
1	27.8%
2	13.0%
3	4.9%

**Table 3:** Personal characteristic, sunscreen knowledge, attitudes, beliefs, and behavior associated with sunscreen use (usually vs. sometimes) (*n* = 151).

Variable	Odds Ratio (95% CI)	*p*-Value
Age (by year)	1.03 (1.01, 1.06)	0.005
Gender, (male vs. female)	0.34 (0.13, 0.93)	0.035
Race (Others vs. Caucasian/White)	1.31 (0.43, 4.01)	0.635
Residential State (Florida vs. California)	0.95 (0.45, 2.04)	0.903
Education (non-college graduated vs. college graduated)	0.91 (0.38, 2.14)	0.824
Sunscreen knowledge score (from 0 to 7)	1.51 (1.06, 2.16)	0.024
Skin cancer knowledge score (from 0 to 6)	0.94 (0.64, 1.38)	0.748
Negative sunscreen beliefs score (somewhat agree vs. somewhat disagree)	1.01 (0.32, 3.18)	0.983
General sunscreen beliefs score (somewhat agree vs. somewhat disagree)	4.03 (0.52, 31.35)	0.182
Sunscreen apply count (from 1 to 9)	1.08 (0.91, 1.27)	0.370

**Table 4:** Personal characteristics, sunscreen knowledge, attitudes, beliefs, and behavior associated with sunburn (yes vs. no) (*n* = 151).

Variable	Odds Ratio (95% CI)	*p*-Value
Age (by year)	0.94 (0.91, 0.96)	<0.0001
Gender, (male vs. female)	1.40 (0.44, 4.49)	0.569
Race (Others vs. Caucasian/White)	0.22 (0.06, 0.76)	0.017
Residential State (Florida vs. California)	0.75 (0.33, 1.74)	0.508
Education (non-college graduated vs. college graduated)	1.56 (0.61, 4.00)	0.354
Sunscreen knowledge score (from 0 to 7)	1.07 (0.73, 1.55)	0.735
Skin cancer knowledge score (from 0 to 6)	0.76 (0.5, 1.15)	0.189
Negative sunscreen beliefs score (somewhat agree vs. somewhat disagree)	2.14 (0.53, 8.59)	0.283
General sunscreen beliefs score (somewhat agree vs. somewhat disagree)	0.19 (0.03, 1.32)	0.092
Sun-protection behavior score (usually vs. sometimes)	0.33 (0.13, 0.82)	0.017
Sunscreen apply count (from 1 to 9)	1.13 (0.94, 1.34)	0.190

## Data Availability

Data and the survey codebook will be made available upon request in a de-identified form. Those requesting use of the data will need to submit a proposal to the investigators to ensure that the proposed analyses are appropriate for the data and do not duplicate previous analyses or publications.

## Supplementary Materials

The following supporting information can be downloaded at: https://www.mdpi.com/article/10.3390/ijerph19052677/s1, File S1: Sunscreen Survey 2019.
