# Supplementary material for: Development of a Survey of Sunscreen Use and Attitudes among Adults in Two Coastal States, 2019
Source: Int J Environ Res Public Health. 2022 Feb 25;19(5):2677. doi: 10.3390/ijerph19052677 (PMC8910541; doi:10.3390/ijerph19052677)
Supplement: Supplementary file 1 [file ijerph-19-02677-s001.zip › ijerph-1548744-supplementary.pdf]

Sunscreen Survey

1. Sun Protection Habits

When you are outdoors in the sun, in warm weather, how often do you...

	Never (1)	Rarely (2)	Sometimes (3)	Usually (4)	Always (5)
Wear a shirt with sleeves (1)	❍	❍	❍	❍	❍
Wear sunglasses (2)	❍	❍	❍	❍	❍
Stay in the shade or under an umbrella (3)	❍	❍	❍	❍	❍
Use sunscreen (4)	❍	❍	❍	❍	❍
Limit your time in the sun during midday hours (5)	❍	❍	❍	❍	❍
Wear a hat (6)	❍	❍	❍	❍	❍

When do you usually put on sunscreen?

- ❍ Before going outside (1)
- ❍ When I get outside (2)
- ❍ After being outside for 30 minutes or more (3)
- ❍ Never use sunscreen (4)

How often do you apply sunscreen before engaging in the following outdoor activities? If you do not engage in those activities, select N/A.

	Never (1)	Rarely (2)	Sometimes (3)	Usually (4)	Always (5)	N/A
Swimming (1)	❍	❍	❍	❍	❍	❍
Snorkeling (2)	❍	❍	❍	❍	❍	❍
Surfing (3)	❍	❍	❍	❍	❍	❍
Paddling (4)	❍	❍	❍	❍	❍	❍
Other Water Sports (5)	❍	❍	❍	❍	❍	❍
Beach activities and leisure or relaxation (6)	❍	❍	❍	❍	❍	❍

2. Sun Exposure

In the past year, did you get a sunburn - or reddening of skin - from being out in the sun?

- ❍ No (1)
- ❍ Yes, once (2)
- ❍ Yes, twice (3)
- ❍ Yes, more than twice (4)

3. Sunscreen Purchasing

Please rate the importance of the different features you consider when purchasing sunscreen. Rate each feature from (1) Unimportant to (5) Critical.

	Unimportant (1)	Slightly Important (2)	Moderately Important (3)	Very Important (4)	Critical (5)
Price (1)	❍	❍	❍	❍	❍
Water Resistant (2)	❍	❍	❍	❍	❍
Broad Spectrum (3)	❍	❍	❍	❍	❍
SPF 50+ (4)	❍	❍	❍	❍	❍
SPF 15+ (5)	❍	❍	❍	❍	❍
Ingredients (6)	❍	❍	❍	❍	❍
Sunscreen that can be sprayed (7)	❍	❍	❍	❍	❍
Other product features (8)	❍	❍	❍	❍	❍

Out of the features listed in the previous question, which is the most important to you when purchasing sunscreen?

- ❍ Price (1)
- ❍ Water Resistant (2)
- ❍ Broad Spectrum (3)
- ❍ SPF 50+ (4)
- ❍ SPF 15+ (5)
- ❍ Ingredients (6)
- ❍ Sunscreen that can be sprayed (7)
- ❍ Other product features, please describe: (9) ____________________

What brand of sunscreen do you most often use? (e.g. Banana Boat Sport, Neutrogena Ultra Sheer)

____

What type of sunscreen do you most often use?

- ❍ Lotion (1)
- ❍ Spray (2)
- ❍ Stick (3)
- ❍ Oil (4)
- ❍ Gel (5)
- ❍ Cream (6)

What is the Sun Protection Factor (SPF) number of your preferred sunscreen? (e.g. SPF 8, SPF 15, SPF 50) ____

Is the sunscreen that you most often use a chemical sunscreen, with active ingredients like avobenzone, octinoxate, and/or oxybenzone?

- ❍ No (1)
- ❍ Yes (2)
- ❍ Not sure (3)

Is the sunscreen that you most often use a mineral sunscreen, with active ingredients like titanium dioxide and/or zinc oxide?

- ❍ No (1)
- ❍ Yes (2)
- ❍ Not sure (3)

Where do you typically purchase sunscreen?

- ❑ Grocery Store (1)
- ❑ Drug Store, like Walgreens or Longs (2)
- ❑ Convenience Store, like ABC Stores or 7-11 (3)
- ❑ Online Marketplace, like Amazon (4)
- ❑ Store Website, like CVS.com (5)
- ❑ Manufacturer or Product Website (6)
- ❑ Other, please describe: (7) ____________________

4. Knowledge

Read each statement and select if you think it is true or false.

	TRUE (1)	FALSE (2)	DON'T KNOW (3)
To work best, sunscreen needs a half hour to be absorbed by the skin. (1)	❍	❍	❍
A Sun Protection Factor (SPF) of 15 or higher means you can stay outside for 3 hours without worrying about getting a burn. (2)	❍	❍	❍
The best type of sunscreen protects you against "UVA" rays only. (3)	❍	❍	❍
Sunscreen should be re-applied every 2 hours if you are outside for a long time. (4)	❍	❍	❍
Sun exposure in childhood increases the chances of getting melanoma as an adult. (5)	❍	❍	❍
People with dark skin cannot get melanoma. (6)	❍	❍	❍
A sunburn is painful, but really not harmful in the long run. (7)	❍	❍	❍

Read each statement and select if you think it is true or false.

	TRUE (1)	FALSE (2)	DON'T KNOW (3)
There is no need to worry about getting skin cancer if you are exposed to the sun for ten to twenty minutes. (1)	❍	❍	❍
The sun is the strongest when your shadow is short. (2)	❍	❍	❍
People with light hair and light skin are at a greater risk for melanoma. (3)	❍	❍	❍
On cloudy days, you don't need to worry about sun. (4)	❍	❍	❍
Sun exposure when you are young increases your chances of getting melanoma. (5)	❍	❍	❍
Wet skin allows more ultraviolet rays to penetrate the outer, protective layers of skin. (6)	❍	❍	❍
Children should be protected from severe and/or blistering sunburns due to the greatly increased risk of melanoma later in life. (7)	❍	❍	❍

'Broad Spectrum' means the sunscreen product protects against:
- ❍ UVA rays (1)
- ❍ UVB rays (2)
- ❍ Both UVA and UVB rays (3)
- ❍ Neither type. It refers to something else altogether. (4)
- ❍ Don't know (5)

In order to reduce your risk of skin cancer, which of the following sunscreen products should you use?
- ❍ A minimum of SPF 8 & UVB Protection (1)
- ❍ A minimum of SPF 10 & Broad Spectrum (2)
- ❍ A minimum of SPF 15 & Broad Spectrum (3)
- ❍ A minimum of SPF 30 & UVA Protection (4)
- ❍ Don't know (5)

5. Attitudes and Beliefs

**Please rate the following statements from (1) Strongly Disagree to (5) Strongly Agree.

	Strongly Disagree (1)	Somewhat Disagree (2)	Neutral (3)	Somewhat Agree (4)	Strongly Agree (5)
Sunscreen ingredients are toxic. (1)	❍	❍	❍	❍	❍
Most sunscreen is full of harmful chemicals. (2)	❍	❍	❍	❍	❍
Sunscreen isn’t safe to use. (3)	❍	❍	❍	❍	❍
Sunscreens cause damage to coral reefs. (4)	❍	❍	❍	❍	❍
Mineral sunscreen (with ingredients like zinc oxide) provides better protection from the sun. (5)	❍	❍	❍	❍	❍
Chemical sunscreen (with ingredients like oxybenzone or octinoxate) are bad for marine life. (6)	❍	❍	❍	❍	❍

Please rate the following statements from (1) Strongly Disagree to (5) Strongly Agree.

	Strongly Disagree (1)	Somewhat Disagree (2)	Neutral (3)	Somewhat Agree (4)	Strongly Agree (5)
People are more attractive if they have a tan. (1)	❍	❍	❍	❍	❍
Sunscreen lotions are too expensive. (2)	❍	❍	❍	❍	❍
Remembering to carry sunscreen when you go out is inconvenient. (3)	❍	❍	❍	❍	❍
You find it difficult to protect yourself from the sun. (4)	❍	❍	❍	❍	❍
There are so many different recommendations about preventing skin cancer that it's hard to know which ones to follow. (5)	❍	❍	❍	❍	❍
Sunscreen has an unpleasant smell. (6)	❍	❍	❍	❍	❍
It is a hassle to reapply sunscreen. (7)	❍	❍	❍	❍	❍
You're concerned about the safety of ingredients in sunscreen. (8)	❍	❍	❍	❍	❍

6. Awareness of the Sunscreen Ban

Have you heard about the law passed by the Hawai'i State Legislature banning the sale and distribution of sunscreens containing oxybenzone or octinoxate in the state?

❍ No (1)

❍ Yes (2)

❍ Not sure (3)

Read each statement and select if you think it is true or false.

	TRUE (1)	FALSE (2)	DON'T KNOW (3)
It is against the law for stores to sell sunscreen containing oxybenzone or octinoxate in Hawaii. (1)	❍	❍	❍
It is against the law for consumers in Hawaii to purchase sunscreen containing oxybenzone or octinoxate on the internet. (2)	❍	❍	❍
Those visiting Hawaii can bring sunscreen containing oxybenzone or octinoxate with them. (3)	❍	❍	❍

7. Background Information

What is your ZIP code: ________________

How long have you lived in this ZIP Code?
❍ Less than one year (1)
❍ Between one and two years (2)
❍ Between three and five years (3)
❍ Between six and ten years (4)
❍ More than ten years (5)

What is your date of birth? (MM/DD/YYYY) ______________________

What ethnic group do you most identify with?
❍ Caucasian/Non-Hispanic White (1)
❍ Asian (2)
❍ American Indian or Alaskan Native (3)
❍ Black/African American (4)
❍ Latino (5)
❍ Native Hawaiian or other Pacific Islander (6)
❍ Other, please specify: (8) ____________________

What is your highest level of education?
❍ Did not complete high school (1)
❍ High school graduate (2)
❍ Some college, trade, or vocational school (3)
❍ Graduated from a 4 year college (4)
❍ Post-graduation education (5)
